# Investigation of Freezing and Freeze-Drying for Preserving and Re-Using a Whole Microbial Cheese Community

**DOI:** 10.3390/foods13121809

**Published:** 2024-06-08

**Authors:** Wenfan Cao, Stéphanie Passot, Françoise Irlinger, Fernanda Fonseca

**Affiliations:** INRAE, AgroParisTech, UMR SayFood, Université Paris-Saclay, F-911230 Palaiseau, France; wenfancao@gmail.com (W.C.); stephanie.passot@inrae.fr (S.P.); francoise.irlinger@inrae.fr (F.I.)

**Keywords:** microbiota, ecosystem, stabilization, glass transition, cheese ripening, ripening bacteria, yeasts, lactic acid bacteria

## Abstract

Preserving microbial ecosystems obtained from traditional cheese-making processes is crucial to safeguarding the biodiversity of microbial cheese communities and thus ensuring that the high flavor quality of traditional cheeses is maintained. Few protocols have been proposed for the long-term storage of microbial consortia. This work aimed to develop preservation methods to stabilize the entire microbial community in smear-ripened cheese without multiplication or isolation. A simplified microbial community, capable of reproducing the metabolic pattern of cheese maturation, was used in three independent cheese productions. Cheese samples were taken before and after the ripening step, mixed with maltodextrin or saline solution, and subjected to different stabilization conditions including freezing and freeze-drying, followed by 1 month of storage. Microbial survival was quantified using the colony-forming unit assay. Differential scanning calorimetry was used to relate the physical events occurring within the samples to the microbial storage stability. Freezing at −80 °C resulted in the lowest loss of culturability (<0.8 log unit), followed by freezing at −20 °C and freeze-drying. The ripening bacteria appeared as the most sensitive microorganisms within the community. Moreover, a successful cheese production using the best-stabilized community showed the possibility of preserving and re-using an entire microbial community of interest.

## 1. Introduction

Cheese is one of the oldest fermented foods created by humankind. However, cheese production practices have evolved to adapt to market trends and meet the health requirements imposed by public authorities (the addition of the step of milk heat treatment). The obligation to bring production facilities in line with regulatory standards has resulted in a loss of microbial load and diversity in the milk. Most cheeses, whether made from raw or pasteurized milk, are therefore inoculated with ready-to-be-used starters or ripening cultures produced by dedicated food companies. Isolated pure microorganisms are multiplied by fermentation, then concentrated and stabilized by freezing or freeze-drying [1]. Production and stabilization conditions must be optimized for each microorganism, even species or strains, due to the variability in their survival rate. Most starters and ripening cultures available on the market represent a limited variety of species. Furthermore, this ex situ conservation mode facilitates access to the strains but does not preserve all of the diversity of a population. Moreover, the genetic material stored in banks suffers from an evolution halt because the natural selection and adaptation process is prevented [2]. The strains no longer undergo the natural selection pressure of their original environment and are no longer capable of adapting to its changes [3]. They become less and less competitive concerning the microbial communities originating from the traditional cheese-making practices. Therefore, the challenge is to design a new approach to manage microbial genetic resources. A promising alternative would be stabilizing and storing a whole cheese microbiota directly sampled from its original environment without prior isolation. The preservation of the ecosystems’ viability and microbial functionality has been scarcely reported. Published work on ecosystem preservation mainly concerns fecal microbiota [4,5,6,7,8], environmental ecosystems (e.g., soil and water) [9,10,11], microbial communities with biotechnological value (e.g., methanotrophic or denitrifying functions) [12,13,14], and kefir grains [15,16,17]. Many studies focus on preserving DNA or RNA to identify microorganisms in the community, while some works report on preserving technological functionality. The preservation protocols also vary in terms of sample composition. They either include the microbial community and the physical surroundings or the microorganisms are separated (e.g., by filtration) from the matrix. Moreover, different molecules such as sugars (e.g., sucrose, trehalose), polymers (e.g., maltodextrin), and an antioxidant (e.g., sodium ascorbate) [18,19,20,21,22,23] ] are usually added before freezing and freeze-drying to counterbalance the stresses induced by stabilization processes.

The challenges encountered in stabilizing ecosystems compared to isolated pure strains can be explained by their high degree of biodiversity (i.e., different genera, species, and strains). Each microorganism contributing to the ecosystem’s functionality exhibits variable resistance to the stabilization processes. To the best of our knowledge, there is very little research on cheese ecosystem preservation [24], the viability (or culturability) recovery of microorganisms within this matrix, and its use as a vector for microbial reseeding. Developing stabilization methods to preserve whole cheese ecosystems, in which microorganisms have already adapted to each other and the technological context of production, appears as a promising alternative to improving and diversifying cheese products.

In this context, the main goal of this study was thus to evaluate the feasibility of developing a stabilization and storage method for preserving a microbial ecosystem directly in the cheese, using processes conventionally employed for stabilizing starters (freezing and freeze-drying). We specifically intended to evaluate the culturability recovery of each microorganism within the microbial ecosystem. The ability to use a stabilized cheese microbial community to inoculate a new cheese production (instead of using isolated pure microorganism cultures) is also considered.

With this purpose, a model microbial consortium was used to produce a surface-ripened soft cheese at the laboratory scale. The culturability recovery of microorganisms in a cheese ecosystem was quantified for two maturation states (i.e., fresh and ripened cheese) before and after freezing (at −20 °C and −80 °C), freeze-drying, and storage. Frozen and freeze-dried cheese samples were characterized by differential scanning calorimetry to identify a potential correlation between physical events (such as the glass transition temperature) and the storage stability of the microorganisms. Finally, one test cheese production was performed based on the inoculation of the best-stabilized microbial ecosystem from this study. The evolution of the ecosystem microbial composition and the desired technological functionalities (pH and color) were compared to the reference cheese production using inoculation of isolated pure strains.

## 2. Materials and Methods

**Figure 1:**
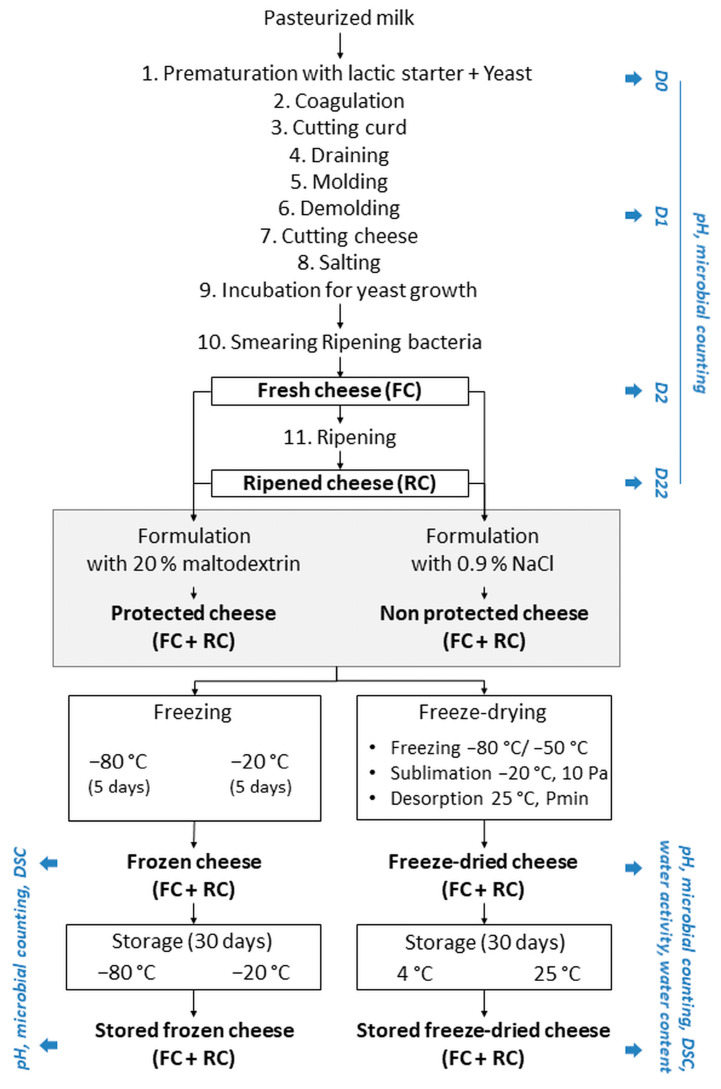
Experimental approach used in the present study. P: pressure; DSC: differential scanning calorimetry.

The experimental approach used in this study is presented in Figure 1. It shows the main stages in cheese production, the treatment options for fresh and ripened cheese samples before stabilization, the different stabilization methods, and the storage conditions. The microbiological and physical analyses carried out throughout the experiment are also mentioned. Three series of experiments from the cheese production to the storage steps were performed (3 biological replicates). The produced cheese was a model of surface-ripened soft cheese, measuring 50 mm in diameter and 15 mm in height.

### 2.1. Strains

The model microbial consortium was composed of ten microorganisms belonging to nine species, which were originally isolated from the following cheeses [25]: two starter cultures assigned to *Lactococcus lactis* subsp. *lactis* S3+ and its protease-negative variant S3-, and eight ripening cultures containing five bacteria and three yeasts. The list of microorganisms used in the present study is available in Table 1. Samples of each microbial strain were stored in cryotubes at −80 °C.

### 2.2. Preparation of Microbial Inocula for Cheese Production

Culture media used to prepare the cheese microbial inocula were sterilized at 121 °C for 15 min, except for the reconstituted skim milk (110 °C for 30 min).

#### 2.2.1. Starters and Ripening Yeast (Inoculation of Milk)

The starters were individually prepared through a three-step preculture in static anaerobic conditions at 30 °C. First, a small amount of the frozen stock culture was sampled using a sterile loop to inoculate 6 mL of sterile M17 medium (Biokar Diagnostics, Beauvais, France). After 24 h of incubation, 200 µL was used to inoculate 20 mL of reconstituted skim milk (Difco^TM^, Difco Laboratories, Le Pont de Claix, France). After another 24 h, 420 µL of *L. lactis* S3+ culture and 8.4 mL of *L. lactis* S3- culture were inoculated into 14 mL and 280 mL of reconstituted skim milk (Difco^TM^, Difco Laboratories, Le Pont de Claix, France), respectively. This third preculture was then incubated for 18 h and directly used to produce cheese.

The ripening yeasts were individually prepared by first growing through a two-step preculture, then harvesting and suspending cells in saline solution (9 g.L^−1^ of NaCl) and storing them at 4 °C until use for cheese production. The preculture was performed in sterile potato dextrose broth (PDB) (Difco^TM^, Difco Laboratories, Le Pont de Claix, France) at 25 °C on a rotary shaker (150 rpm). First, a small amount of the frozen stock culture was sampled using a sterile loop to inoculate 10 mL of PDB. After 48 h of incubation, 900 µL was used to inoculate 90 mL of PDB and further incubated for 24 h for cell collection.

*G. candidum* was collected by vacuum filtering 60 mL of the second preculture (cellulose acetate 0.45 µm filter, Sartorius), and the cell pellet was resuspended in 12.6 mL of sterile saline solution. The concentration of *G. candidum* was approximately 10^7^ CFU.mL^−1^. For *K. lactis* and *D. hansenii*, the cell concentration of the second preculture was estimated by optical density measurement at 600 nm. Then, an appropriate volume of the second preculture was centrifuged at 4500× *g* for 10 min at 4 °C (Centrifugre 5804 R model, Eppendorf, Hamburg, Germany) to obtain 10^9^ CFU of yeast in the pellet. The cell pellet was resuspended in 5 mL of sterile saline solution.

#### 2.2.2. Ripening Bacteria (Inoculation through the Smearing Procedure of Cheese)

Similar to yeast inocula preparation, ripening bacteria were first individually prepared with a two-step preculture in brain heart infusion (BHI) broth (Biokar Diagnostics, Beauvais, France) at 25 °C on a rotary shaker (150 rpm), and then cells were collected for cheese inoculation. Regarding the preculture, the purity of the frozen stock culture was verified using the isolation procedure on BHI agar Petri dishes. Then, one bacterial colony was inoculated in 10 mL of BHI broth. After 48 h of incubation, 600 µL was inoculated into 60 mL of BHI broth and further incubated for 24 h for cell collection.

The cell concentration of the second culture was estimated by optical density measurement at 600 nm. Then, an appropriate volume of the second preculture was centrifuged at 4500× *g* for 10 min at 4 °C (Centrifuge 5804 R model, Eppendorf, Hamburg, Germany) to obtain 3 × 10^10^ CFU of bacteria in the pellet. The cell pellet was resuspended in 3 mL of sterile saline solution and kept at 4 °C. The smearing solution was obtained by adding 500 µL of each bacterial suspension in 47.5 mL of sterile saline solution and then kept at 4 °C until smearing onto the cheese before ripening.

### 2.3. Experimental Cheese Production

Three batches of smear-ripened cheeses were produced from commercial pasteurized milk (Alsace Lait, Hoerdt, France) at a laboratory scale under aseptic conditions as previously described by [25,26], except that cheese production was performed with 14 L of milk instead of 90 L and 120 L, respectively. Forty experimental cheeses (25 ± 1 g each) were produced during a single manufacturing run. Cheese samples were collected on days 0, 1, 2, and 22 (D0, D1, D2, and D22). Cheese collected on D2 after salting, incubation for yeast growth, and smearing of ripening bacteria corresponded to the “fresh cheese (FC)”. The five ripening bacteria were smeared onto the surface of the cheese at a rate of 2 × 10^5^ CFU.g^−1^. The smeared cheeses were ripened for three weeks at 17 °C and 97% relative humidity in sterile crystallizing basins and collected on D22 as the “ripened cheese (RC)” (Figure 1).

All handling was carried out under a laminar flow hood to avoid contamination. Materials in direct contact with milk and milk products and the solutions used were sterilized at 120 °C for 30 min. Incubation containers and lids were disinfected by UV for 30 min.

Throughout the cheese manufacturing process, microbiological analyses and pH measurements were carried out to ensure that the process was running smoothly.

#### 2.3.1. Day 0 of Cheese Production

Commercial pasteurized full-fat milk and semi-skimmed milk (Alsace Lait, Hoerdt, France) were mixed to standardize the milk fat content at 29 g.L^−1^ and heated from 4 °C to 34 °C. The lactic starter and yeast suspensions, along with 14 mL of filter-sterilized CaCl_2_ solution (10% *w*/*v*), were added to the milk when the temperature reached 20 °C. When the milk pH reached 6.3, 4.2 mL of rennet solution (555 mg.L^−1^ of chymosin (Chr. Hansen, Arpajon, France)) was added, allowing milk coagulation in 20 min. After 40 min of hardening, the curd was cut and left to rest for 5 min; it was then stirred for 5 min at 10 rpm and left to rest again for another 5 min. Following this, 5.6 L of whey was removed before molding. The curd was distributed alternatively in two circular molds (diameter of 20 cm and height of 15 cm). During draining, the molds were incubated at 22 °C for 22.5 h and inverted three times (at 30 min, 2.5 h, and 6 h after molding, under a laminar flow hood).

#### 2.3.2. Day 1 of Cheese Production

After 22.5 h of draining, the cheeses were demolded, inverted, and left draining for another 2 h. The final height of the cheeses was approximately 3 cm. The cheeses were then cut into smaller sizes (diameter of 5 cm and height of 1.5 cm) using a circular cookie cutter and a knife. Approximately 40 cheeses of 25 g were obtained. They were then immersed for 3 min in 5 L of sterile brine solution (270 g.L^−1^ of NaCl) at 14 °C, resulting in a final salt concentration of ~1.5%. After a short draining to remove excess brine solution, the cheeses were transferred into sterile crystallizing basins (diameter of 13.5 cm, 4 cheeses per basin) and incubated for 24 h at 25 °C and 93% relative humidity (RH), facilitating the growth of yeasts and the pH increase in the cheese.

#### 2.3.3. Day 2 of Cheese Production

Following the incubation of salted cheese, a volume of 300 µL of the smearing solution was spread on the cheese surface with a sterilized paintbrush, leading to an inoculation level of 1.1 × 10^6^ CFU.g^−1^ of cheese. These so-called FC samples were transferred into sterile crystallizing basins, and then ripened at 17 °C and 97% RH for 20 days until D22, giving the RC.

### 2.4. Experimental Design for Cheese Stabilization and Storage

The FC and RC collected on D2 and D22, respectively, were blended at a 1:1 *w*/*w* ratio either with sterile saline solution (9 g.L^−1^ of NaCl) or with sterile protective solution (200 g.L^−1^ of maltodextrin (Glucidex^®^ 12, Roquette, Lestrem, France)). These two solutions were previously sterilized at 120 °C for 15 min. The mixtures were homogenized at 15,000 rpm for 5 min using an Ultra Turrax^®^ homogenizer (T-25-digital model equipped with a S25N-25F model stem, IKA, Staufen, Germany). The blended cheese suspensions were distributed in round aluminum cups (63 mm in diameter and 18 mm in height, VWR, Rosny-sous-Bois, France) with 20 g per cup.

Freezing and freeze-drying were applied to stabilize the cheese. The cups of cheese designated for freezing were covered with aluminum foil before freezing. The freezing was performed at −80 °C and −20 °C for 5 days and the frozen samples were stored at their freezing temperature for 1 month. The cups of cheeses designated for freeze-drying were first covered and frozen at −80 °C. They were then transferred to pre-cooled shelves at −50 °C in a pilot-scale freeze-dryer (REVO model, Millrock Technology, Kingston, NY, USA). After a holding step of 1 h at −50 °C, the chamber pressure was decreased to 10 Pa, and the shelf temperature was increased from −50 °C to −20 °C at a heating rate of 0.25 °C.min^−1^ to initiate sublimation. After 60 h of sublimation, the shelf temperature was increased to 25 °C at 0.25 °C.min^−1^. After 12 h of desorption, the vacuum was broken by injecting air. The freeze-dried cheeses were vacuum-packed in multi-layer aluminum bags and stored at −80 °C, 4 °C, and 25 °C for 1 month. In addition, the cheeses were weighed before and after freeze-drying to determine the water loss during the process, which is essential for later rehydration.

### 2.5. Microbial Analysis (Culturability and pH Measurements)

Throughout the cheese manufacturing process, cheese samples were harvested aseptically on D0, D1, D2, and D22 to monitor pH and the evolution of the microbial community. After each preservation process and 1 month of storage, sampling was also carried out to assess the culturability of each microorganism, which is the microbial strain’s ability to grow on a nutrient-rich medium (in colony-forming units).

One g of solid samples was added to 9 mL of sterile saline solution and blended using an Ultra Turrax^®^ homogenizer (T-25-digital model equipped with a S25N-25F model stem, IKA, Staufen, Germany) to form a suspension. Cheese samples collected during manufacturing were treated at 15,000 rpm for 3 min. Frozen cheese samples were crushed, without thawing, at 15,000 rpm for 30 s. Freeze-dried cheese samples were firstly reconstituted by adding saline solution to form a suspension corresponding to a decimal dilution of the original sample before freezing. Homogenization with an Ultra Turrax^®^ homogenizer (T-25-digital model equipped with a S25N-25F model stem, IKA, Staufen, Germany) at 6000 rpm for 3 min was also performed to promote hydration.

Serial dilutions of suspensions were performed and plated in duplicate on agar plates. Three selective culture media were used. Ripening bacteria were counted on brain heart infusion (BHI) agar (Biokar Diagnostics, Beauvais, France) supplemented with amphotericin B (50 mg.L^−1^, Sigma-Aldrich^®^, Merck KGaA, Darmstadt, Germany) after being incubated at 25 °C for 72 h and then exposed to light for 4 days. Yeasts and lactic bacteria were counted on yeast extract glucose chloramphenicol (YEGC) (Biokar Diagnostics, Beauvais, France) supplemented with 2,3,5-Triphenyltetrazolium chloride (TTC, 10 mg.L^−1^) (Sigma-Aldrich^®^, Merck KGaA, Darmstadt, Germany) and on Man–Rogosa–Sharpe medium (MRS) (Biokar Diagnostics, Beauvais, France) supplemented with amphotericin B (50 mg.L^−1^, Sigma-Aldrich^®^, Merck KGaA, Darmstadt, Germany), respectively. Yeasts were incubated at 25 °C for 48 h and lactic bacteria were incubated in anaerobic conditions at 30 °C for 48 h.

The different ripening bacteria and yeasts can be easily counted on these media owing to a distinct morphotype for each species (color and morphological aspects of colonies, Table S1, Supplementary Materials). The yeasts are pigmented red or pink due to the TTC added to the culture medium, while the bacteria are naturally pigmented. However, it was not possible to differentiate the two strains of *L. lactis* (S3+ and S3−).

The loss of culturability post-stabilization and post-storage were determined for each microorganism of the microbial community by subtracting the logarithmic cell count values before stabilization from those after stabilization and after storage, respectively.

During D0 and the milk prematuration process, pH and temperature were continuously measured. Cheeses sampled at D1 (~20 g) and D2 (~25 g) were crushed to a homogenous paste before pH measurement. For ripened cheeses sampled at D22 (~25 g), pH measurements were performed at the surface and core of the cheese.

### 2.6. Differential Scanning Calorimetry (DSC) of Frozen and Freeze-Dried Samples

Measurements of the physical events occurring following cooling and heating of the samples were performed on two different power compensation DSC types of equipment (Perkin Elmer LLC, Norwalk, CT, USA): a Diamond model equipped with a liquid nitrogen cooling accessory (CryoFill, Perkin Elmer) for the freeze-thawed samples, and a Pyris 1 model equipped with a mechanical cooling system (Intracooler 1P, Perkin Elmer) for the freeze-dried samples. Temperature calibration was conducted using cyclohexane (crystal–crystal transition at −87.1 °C) and mercury (melting point at −38.6 °C) for the Diamond; and cyclohexane (melting point at 6.5 °C), n-octodecane (melting point at 27.8 °C), and indium (melting point at 156.6 °C) for the Pyris 1. About 15–25 mg of the sample was sealed in aluminum pans. Cooling and heating rates of 10 °C.min^−1^ were used. Freeze-thawed samples were scanned following cooling to −120 °C and heating to 25 °C. Freeze-dried samples were heated from −15 to 40 °C, cooled to −15 °C, and heated again to 145 °C. The glass transition temperature of freeze-thawed samples (Tg’) was determined as the temperature at the maximal value of the peak observed in the first derivative of the heat flow curve. No glass transition event could be identified in the freeze-dried samples. Measurements were performed at least in duplicate on each biological replicate.

### 2.7. Water Activity and Water Content Measurements of the Freeze-Dried Cheese Samples

The water activity of samples was measured at 25 °C using an aw meter (labMasteraw model, Novasina, Lachen, Switzerland). The moisture content of samples was measured by the Karl Fisher titration method using a coulometer with a generator electrode with a diaphragm (KF 756 model, Metrohm, Herisau, Switzerland). At least 20 mg of cheese powder was mixed with 2 mL of dry methanol and titrated with the Hydranal reagent (Honeywell Research Chemicals, Seetze, Germany) until the endpoint was reached. Following the freeze-drying process, the cheeses’ water activity and moisture content were found to be less than 0.05 and 5%, respectively.

### 2.8. Cheese Production with Frozen Ecosystems

The frozen cheeses with the highest culturability recovery of the microbial community were used for cheese production to assess the preservation of the community’s functionality (i.e., the ability to inoculate a new cheese production). The cheese production was performed with the same protocol except that the inocula for starters and yeasts and the smearing solution were obtained from stabilized cheese ecosystems (FC and RC frozen in saline solution at −80 °C). The inoculum of starters and yeasts was constructed by adding frozen FC (20 g) in 280 mL of reconstituted skim milk (Difco^TM^, Difco Laboratories, Le Pont de Claix, France). The solution was shaken at 900 rpm for 30 min and then incubated at 30 °C for 16 h. The frozen RC (20 g) was diluted in 30 g of saline water to make a smearing solution. The microbial analyses were also performed on D0, D1, D2, and D22 of the cheese production to monitor the evolution of the microbial community.

The ability to use frozen cheese microbial communities to inoculate a new cheese production was assessed by monitoring pH and microorganisms’ culturability all along the cheese production process. Furthermore, the color of the ripened cheese was qualitatively evaluated.

### 2.9. Statistical Analyses

To compare data concerning the culturability loss of each microorganism following stabilization processes and storage, the nonparametric Kruskal–Wallis and the post hoc Conover Iman tests were performed on medians using XLSTAT 19.6 (Addinsoft, Paris, France) and a significance level of 95% (*p*-value < 0.05).

## 3. Results and Discussion

### 3.1. Effect of the Stabilization Process on the Cheese Microbial Community Composition

**Figure 2:**
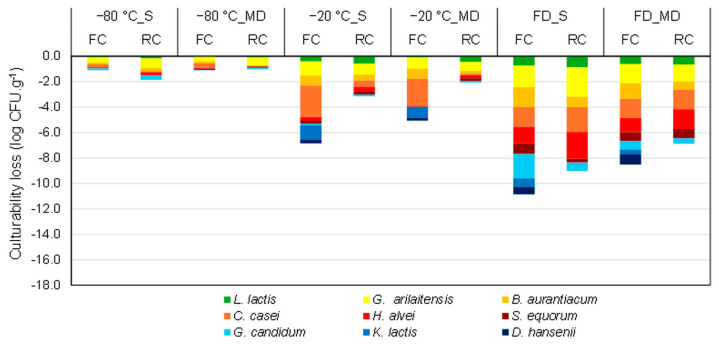
Culturability loss (in log CFU.g^−1^) of cheese ecosystem microorganisms in fresh cheese (FC) and ripened cheese (RC) in the presence of saline solution (S, 0.9% NaCl *w*/*w*) and maltodextrin DE 12 (MD, 20% *w*/*w*) solutions, after freezing at −80 °C and −20 °C, and after freeze-drying (FD). *D. hansenii* and *K. lactis* were not detected in RC samples due to the development of *G. candidum*. The data presented are medians of three biological replicates and two technical replicates.

The current study investigated the effect of different stabilization processes on the microbial community composition of FC and RC ecosystems by measuring the culturability of the different microorganisms before and after stabilization. Figure 2 displays the culturability losses of each microorganism observed after stabilization (freezing at −20 °C and −80 °C, or freeze-drying) with or without protection (maltodextrin or saline solution). The median values shown in Figure 2, as well as the associated interquartile ranges and the results of the statistical analyses, are presented in the Supplementary Material files (Tables S2 and S3, Supplementary Materials). Regardless of the stabilization process, the two ripening yeasts in RC, *D. hansenii* and *K. lactis*, which were already hardly detectable by the plate count method before stabilization, were entirely undetectable afterward. This was likely due to the development of *G. candidium* covering the plate, which interfered with the growth or detection of *D. hansenii* and *K. lactis*. Thus, we cannot draw conclusions regarding the survival rate of those two yeasts in RC.

#### 3.1.1. Stabilization Processes

The entire microbial communities of FC and RC were well preserved during freezing at −80 °C with a culturability loss lower than 0.8 log unit. Applying freezing at −20 °C and freeze-drying resulted in culturability losses that could reach more than 2 log units. When considering an average loss of culturability for all microorganisms, freeze-drying appeared as the least efficient preservation alternative.

Freezing and freeze-drying are widely used techniques to preserve highly concentrated suspensions obtained from axenic cultures of probiotic and lactic acid bacteria [18,27,28]. Freeze-drying is acknowledged to be more stressful than freezing [19,20,21]. In addition to the freezing stress, freeze-drying also brings stress generated from the water removal, especially the desorption of unfrozen water, which can damage membrane lipids and the structure of sensitive proteins [19,20,22,23,29,30].

Most reported studies on ecosystem preservation concern fecal microbiota transplantation. The fecal communities can be well preserved by freezing at −80 °C [5,31,32,33] or by freeze-drying [7,34] without losing therapeutic efficacy. Since a filtration step is always involved in fecal sample preparation, these preservation protocols are often referred to as ex situ methods. They usually include the addition of protective molecules such as glycerol for freezing and a mixture of maltodextrin and trehalose for freeze-drying [5,6]. However, few studies have been carried out on the freezing or freeze-drying of food microbial communities. Bolla et al. [35] investigated the effect of freeze-drying on the culturability of a microbial mixture containing bacterial and yeast strains isolated from kefir grains (*L. kefir*, *L. plantarum*, *L. lactis*, *S. cerevisiae*, and *K. marxianus*) in the presence of PBS buffer, UHT milk, and a milk fermented product. In this ex situ study, milk proved to be the most effective protectant giving the highest survival rates for all microorganisms. Moreover, Mushtaq et al. [24] studied the freezing and storage of Kalari cheese samples at −18 °C and measured the effects on *Lactobacilli*, *Lactococci*, yeasts, and molds. They found such conditions significantly decreased the viability of *Lactobacilli* and *Lactococci*.

#### 3.1.2. Cheese Matrix and Protective Molecules

In the present work, adding maltodextrin before stabilization slightly improved the microbial composition recovery of the cheese ecosystems (after freezing at −20 °C and after freeze-drying), especially for *G. candidum* in FC and *G. arilaitensis* in RC after freeze-drying. Furthermore, when considering freezing at −20 °C, the matrix of RC seemed to provide a better protective ability for *C. casei* and *G. arilaitensis* compared with FC.

Milk has been widely used as a protective molecule for improving the survival rate (viability recovery) after freezing and freeze-drying of a wide variety of individual bacteria, especially lactic acid bacteria [35,36,37,38,39]. More recently, maltodextrin, or a mixture of maltodextrin and sugar, was evidenced as efficient for preserving LAB following freeze-drying [20,37].

The better protective ability provided by RC compared to FC may be ascribed to the matrix’s pH. RC exhibited a pH value of approximately 6.8 compared to 4.8 for the FC. The more acidic environment of FC due to the acids produced by *L. lactis* at the beginning of the cheese production could generate acidic stress, principally affecting, in this work, the survival of *G. arilaitensis*, *B. aurantiacum*, and *C. casei* after freezing. Furthermore, the higher culturability losses observed for these three ripening bacteria in the FC samples compared to the RC samples might also be ascribed to their initial concentration in the samples (Table S4, Supplementary Materials). Being inoculated only on day 2 of the cheese production process, their concentration appeared quite low in FC (between 5.5 and 6.3 log) and significantly increased in RC (between 7.5 and 9.2 log). Some authors reported a beneficial effect of increasing the initial bacterial concentration on the freeze and freeze-drying resistance of the pure culture of bacteria [39,40,41].

#### 3.1.3. Specific Microorganism Sensitivity

Among the microbial community, ripening bacteria appeared as the most sensitive group to stabilization processes, followed by the ripening yeast, while the starter bacterium *L. lactis* exhibited the highest resistance. Several authors have also reported a species-dependent resistance to the preservation processes [42,43,44]. The high resistance of *L. lactis* strains to freezing and freeze-drying previously reported indicated a viability loss lower than 0.5 log units after freeze-drying with a mixture of maltodextrin and trehalose [19] or milk and sucrose [35] as protectants.

Among the ripening bacteria, *S. equorum* and *H. alvei* exhibited the highest resistance, followed by *B. aurantiacum*, and then *C. casei* and *G. arilaitensis*. While there is no reported work on the resistance of *H. alvei*, several works are consistent on the good resistance to freezing of *Staphylococci* in milk frozen at −20 °C and −80 °C [45,46,47]. Moreover, a strain of *S. aureus* maintained approximately 96% culturability after freeze-drying, even without a protectant [48]. *Brevibacterium linens* exhibited 100% survival after freeze-drying in skim milk [49]. Moreover, previous work [42] showed 80% survival rates of *Brevibacterium* (*B. flavum* and *B. lactofermentum*) and *Corynebacterium* (*C. acetoacidophilum* and *C. glutamicum*) species, thus pointing out different resistance among species of the same genus. Similarly, while *G. arilaitensis* exhibits poor resistance to freezing (loss > 1 log unit) and freeze-drying (loss > 2 logs), a 40% survival rate was observed for *A. chlorophenolicus* A6 [50], a close strain that shares a large region of chromosome synteny [51].

Yeast behaved differently depending on the microorganisms. *G. candidum* exhibited the highest resistance to freezing and freeze-drying, similar to a previously reported work using skim milk and 23% trehalose [52]. *D. hansenii* also exhibited high resistance to freezing at −20 °C and −80 °C (loss < 0.5 log units in FC) and freeze-drying with maltodextrin (loss < 1 log unit in FC), in agreement with previous work using skim milk and sodium glutamate as protectants (*D. hansenii* NBRC 15, [53]). Moreover, *K. lactis* CLIB210 presented poor resistance to freezing at −20 °C (loss ~1.4 log units in FC) like its closest relative *K. marxianus* [54]. However, a different *K. marxianus* strain showed a high survival rate after freeze-drying in milk (culturability loss lower than 0.1 log units) [35], thus highlighting the strain specificity.

### 3.2. Evolution of the Composition of the Cheese Microbial Community during Storage in the Frozen and Dehydrated States

**Figure 3:**
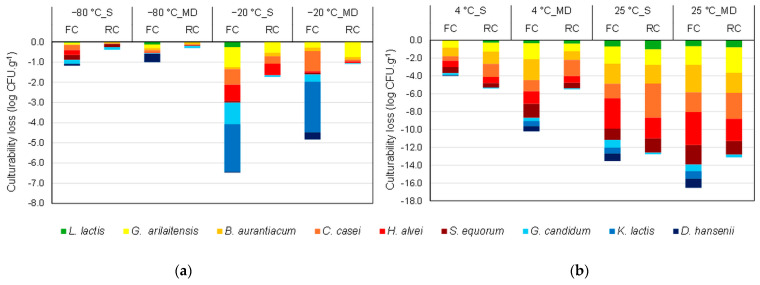
Culturability loss (in log CFU.g^−1^) of cheese ecosystem microorganisms in fresh cheese (FC) and ripened cheese (RC) after 1 month of (**a**) post-freezing storage at −80 °C and −20 °C, and (**b**) post-freeze-drying storage at 4 °C and 25 °C in the presence of saline (S) and maltodextrin (MD) solution. *D. hansenii* and *K. lactis* were not detected in RC samples due to the development of *G. candidum*. The data presented are medians of three biological replicates and two technical replicates.

Cheeses were stored under specific conditions following stabilization by freezing and freeze-drying (Figure 1). The culturability losses after one month of storage were determined and are presented in Figure 3a for post-freezing samples and Figure 3b for post-freeze-drying samples. The median values shown in Figure 3, as well as the associated interquartile ranges and the results of the statistical analyses, are presented in the Supplementary Material files (Tables S5 and S6, Supplementary Materials). The loss of culturability following storage was calculated by subtracting the logarithmic cell count values after storage from those after freezing or freeze-drying (Figure 3).

Regardless of the microorganism and the composition of the matrix (FC or RC, with saline or maltodextrin solution), no loss of culturability was observed after frozen storage at −80 °C (losses lower than 0.26 log units). Increasing the frozen storage temperature raised the culturability losses, particularly when considering the FC samples. Using maltodextrin slightly reduced the culturability losses observed following frozen storage. Moreover, the RC matrix offered the best protection, with losses of culturability lower than 0.8 log units. The protective ability of the RC matrix was remarkable when considering the ripening yeast *G. candidum*.

Considering the storage stability of freeze-dried samples (Figure 3b), storage at 4 °C resulted in losses of culturability lower than 2.4 and 1.9 log units for FC and RC, respectively. Increasing the storage temperature to 25 °C raised the culturability losses to 3.8 log units. Furthermore, adding maltodextrin seemed to have no beneficial effect on the storage stability and the group of ripening bacteria was the most sensitive to freeze-dried storage.

### 3.3. Correlation between the Storage Stability and the Physical Events Observed by DSC

The storage stability of frozen and dehydrated microbial culture products is recognized as being governed by the glass transition temperature of the sample (Tg) and the maintenance of the microorganism within a glassy matrix (i.e., T product < Tg) [18,20,30,55,56]. When the storage temperature is under Tg, the matrix can reach a highly viscous glassy state where molecular mobility is reduced and thus the chemical reactions and microbial inactivation are limited. In contrast, when the storage temperature is above Tg, samples are in a rubbery form, representing an unstable state where the microbial degradation is activated due to the increased molecular mobility.

**Figure 4:**
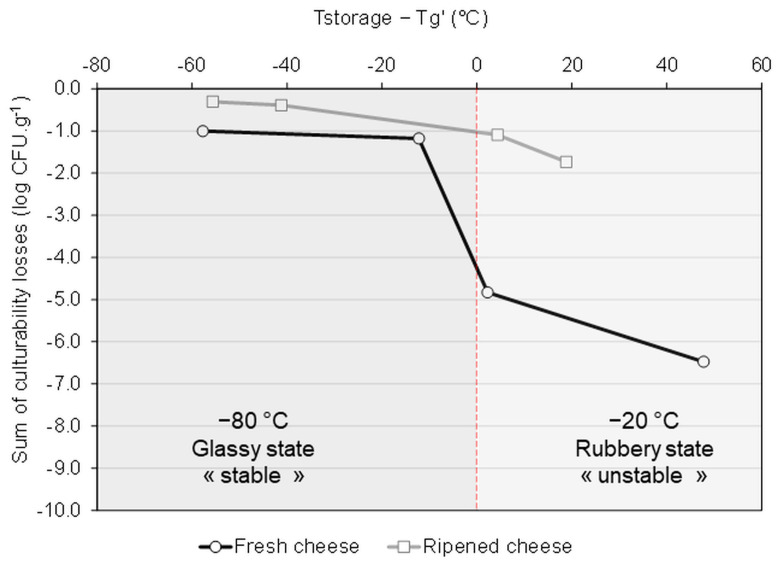
Relationship between the sum of culturability losses of all microorganisms of the cheese ecosystem following 1 month of storage in the frozen state and the temperature difference between the storage temperature and the glass transition temperature of the freeze-concentrated phase (Tstorage—Tg’).

The glass transition temperature of the maximally freeze-concentrated phase (Tg’) of the frozen FC and RC samples was determined by DSC. DSC thermograms (Figure S1, Supplementary Materials) show that the ripening process resulted in a remarkable increase in Tg’ value, which was raised from −68 °C in FC, to −39 °C in RC. The Tg’ value was further increased to −22 °C by adding maltodextrin to the cheese samples. Figure 4 displays the relationship between the sum of culturability losses of microorganisms within the ecosystem following 1 month of storage and the temperature difference values of Tstorage-Tg’. Negative values of Tstorage—Tg’ indicate that the storage of the frozen samples occurred at a temperature lower than the Tg’ value, i.e., that the samples were stored in a stable glassy state. We observed a decrease in the culturability loss when the Tstorage—Tg’ value was negative (storage at −80 °C). The highest culturability losses were observed for the samples stored in the unstable rubbery state (positive values of Tstorage—Tg’, i.e., FC at −20 °C).

The DSC analysis of the freeze-dried cheese samples evidenced several thermal events at temperatures above 5 °C but no glass transition event was detected (Figure S2, Supplementary Materials). Adding maltodextrin or saline solution had no significant effect on the heat flow vs temperature profiles. Instead, some differences were observed between FC and RC. While we cannot unequivocally interpret the complex DSC thermograms, the present work supported the DSC profiles obtained for Emmental cheese [57] in similar steps of the manufacturing process: after the brining and after the ripening. The exothermic peaks recorded on cooling between 5 and 25 °C (Figure S2a, Supplementary Materials) were associated with the crystallization of cheese fat in the cheese samples [57]. Moreover, the overlapping events (several endothermic and one exothermic) recorded during subsequent heating (Figure S2b, Supplementary Materials) were associated with melting and several crystalline reorganizations of cheese fat. Cheese proteins (caseins) cannot be responsible for the thermal transitions observed between 10 and 40 °C [58]. The melting of cheese fat could be associated with the significant culturability losses observed during storage of the freeze-dried samples either at 4 °C or 25 °C.

### 3.4. Cheese-Making and Ripening with Frozen Microbial Communities

After assessing the survival profile of the cheese microbial community under each stabilization and storage condition, the best-preserved cheese ecosystems were selected for functionality analyses via a new cheese-making test.

FC and RC frozen at −80 °C within a saline solution and then stored at −80 °C for one month showed the highest culturability of the entire microbial community and were thus chosen for the cheese-making test. After a ripening period of 20 days (until day 22), the overall microbial population in cheeses produced from the −80 °C frozen ecosystems was like that achieved in the reference production (Table 2). However, the population of two ripening bacteria (i.e., *G. arilaitensis* and *B. aurantiacum*) and two yeasts (i.e., *D. hansenii* and *G. candidum*) were 1–3 log units less than the reference.

Cell counts during the cheese-making showed that *L. lactis* and *K. lactis* came from frozen cheese developed at the expected rate (Table 2). Although *G. candidum* was inoculated at a low quantity, it achieved the reference concentration on D2 of the cheese-making. In contrast, *D. hansenii* was also inoculated at a low concentration, but the reference concentration was not recovered. However, the growth profile of *D. hansenii* was like the reference, suggesting the functionality of this stain is partially maintained. Moreover, with the correct development of *K. lactis* and *G. candidum*, the absence of *D. hansenii* did not appear to impact the deacidification of cheese (Table 2) and the ripening bacteria grew successfully on the cheese surface.

**Figure 5:**
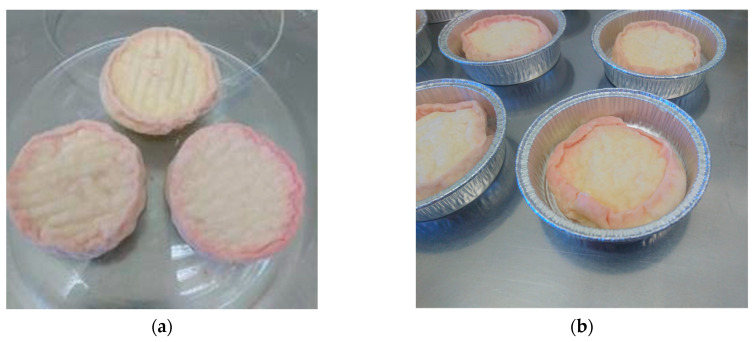

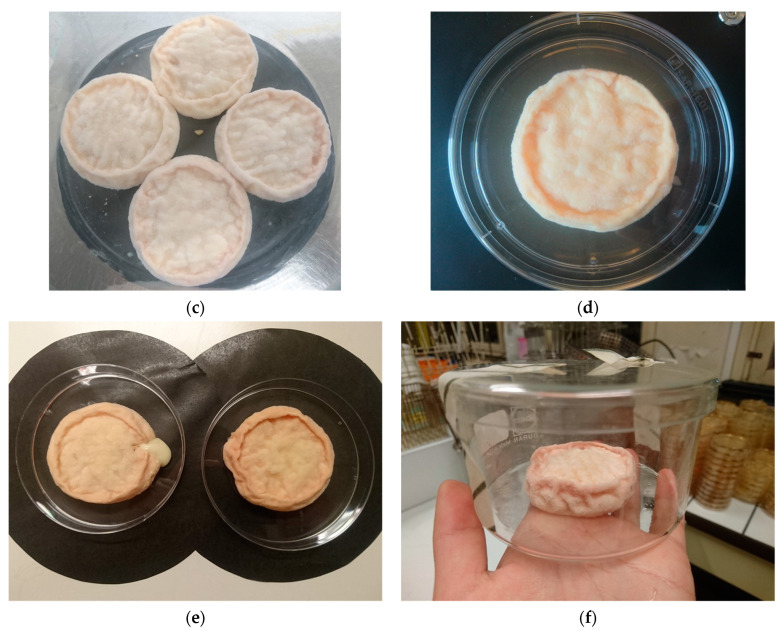
Color comparison between the 20-day (D22) ripened cheese from the reference production (**a**,**b**) and the 20-day (**c**,**d**) and 32-day (D34) (**e**,**f**) ripened cheeses from the test production with frozen ecosystems.

In terms of functionality, apart from pH measurements, a qualitative assessment of the cheese color was performed through visual aspect observation. On D22, the color on the cheese surface was paler than the reference, which agrees with the low cell count results of *G. arilaitensis* and *B. auratiacum* in ripening flora. These two bacteria are the main carotenogenic species responsible for the coloration of smear-ripened cheese [59,60]. However, this color defect was overcome by extending the ripening period to D34 (Figure 5). Together with the cell count results, we assumed that the color defect was due to the low-strain population rather than the functionality defect of frozen bacteria. Moreover, since *D. hansenii* is proven to be promotive for high cheese coloration produced by *B. aurantiacum* [61], the lack of *D. hansenii* growth could also partially explain the fade color observed after 20 days of ripening.

## 4. Conclusions

This work contributed to a better knowledge of the resistance to stabilization processes of each microorganism within a microbial community in two cheese ecosystems: fresh and ripened cheese. The ripening bacteria were the most sensitive, followed by yeast, and among these microorganisms, *G. arilaitensis*, *C. casei*, *H. alvei*, and *K. lactis* were the most sensitive ones. Physical events occurring within the samples appeared to govern the microbial storage stability (i.e., glass transition for frozen samples and fat melting for freeze-dried samples).

Moreover, one cheese production was successfully carried out by inoculating with the best-stabilized fresh and ripened cheeses, thus confirming the possibility of stabilizing the cheese ecosystem obtained during cheese manufacture and reusing the whole ecosystem in future productions. Based on our research, freezing and frozen storage at −80 °C is an effective way to preserve both the culturability and functionality of cheese microbial communities. In this study, the assessment of the ecosystem’s functionality was limited to its ability to reproduce pH and visual aspect evolution following the cheese-making process. A more quantitative evaluation of the organoleptic quality of the cheese (color and aroma profiles, texture, and sensory analysis) could be interesting to perform.

Since using ultra-low temperatures often represents a technological limitation for cheese companies, developing a stabilization protocol involving an intermediate temperature such as -45 °C could be an option. Nevertheless, this will probably require increasing the glass transition temperature of the samples by adding protective molecules. Oligosaccharides exhibiting prebiotic properties offer a promising alternative worthy of further study.

## Figures and Tables

**Table 1:** List of the microbial strains and their origin.

Group	Species	Strain	Origin
Starter	*Lactococcus lactis* subsp. *lactis*	S3+	SayFood collection, INRAE, Palaiseau, France
	*Lactococcus lactis* subsp. *lactis*	S3-	SayFood collection, INRAE, Palaiseau, France
Ripening Bacteria	*Glutamicibacter arilaitensis* *	Re117	Institut Pasteur collection, Paris, France
	*Brevibacterium aurantiacum*	ATCC 9174	American Type Culture Collection, Rockville, MD, USA
	*Corynebacterium casei*	2M01	ABTE collection, UNICAEN, Caen, France
	*Hafnia alvei*	GB001	SayFood collection, INRAE, Palaiseau, France
	*Staphylococcus equorum*	Mu2	SayFood collection, INRAE, Palaiseau, France
Ripening Yeasts	*Kluyveromyces lactis*	CLIB210	SayFood collection, INRAE, Palaiseau, France
	*Debaryomyces hansenii*	304	SayFood collection, INRAE, Palaiseau, France
	*Geotrichum candidum*	ATCC 204307	American Type Culture Collection, Rockville, MD, USA

* Formerly Arthrobacter arilaitensis.

**Table 2:** Evolution of pH and cell concentration of the different microorganisms (in CFU.g^−1^) during the cheese production process using frozen ecosystems as inocula (test production) compared to the reference production (the biological replicate from which the frozen ecosystems originated).

	Test Production (Frozen Ecosystems)				Reference Production			
	Day 0	Day 1	Day 2	Day 22	Day 0	Day 1	Day 2	Day 22
pH	6.57	4.55	4.57	7.09	6.69	4.66	4.45	6.99
*G. arilaitensis*	-	-	6.0 × 10^2^	1.9 × 10^5^	-	-	6.9 × 10^5^	5.5 × 10^7^
*B. aurantiacum*	-	-	1.1 × 10^4^	1.4 × 10^6^	-	-	3.4 × 10^5^	4.5 × 10^7^
*C. casei*	-	-	1.4 × 10^6^	1.6 × 10^9^	-	-	1.0 × 10^6^	1.5 × 10^9^
*H. alvei*	-	-	5.5 × 10^5^	1.8 × 10^8^	-	-	1.0 × 10^6^	1.1 × 10^8^
*S. equorum*	-	-	3.2 × 10^5^	6.3 × 10^7^	-	-	1.4 × 10^6^	1.3 × 10^8^
*K. lactis*	1.8 × 10^4^	5.9 × 10^6^	1.4 × 10^7^	ND	5.2 × 10^4^	3.0 × 10^6^	8.3 × 10^6^	ND
*D. hansenii*	8.8 × 10^3^	4.3 × 10^4^	3.5 × 10^5^	2.0 × 10^5^	2.8 × 10^5^	3.06 × 10^6^	1.3 × 10^7^	1.0 × 10^6^
*G. candidum*	1.0 × 10^1^	2.9 × 10^3^	2.0 × 10^6^	8.6 × 10^7^	3.0 × 10^2^	1.1 × 10^5^	8.8 × 10^4^	1.1 × 10^8^
*L. lactis* S3+/S3-	1.4 × 10^7^	5.6 × 10^9^	2.1 × 10^9^	1.0 × 10^8^	1.1 × 10^7^	7.5 × 10^9^	5.5 × 10^9^	4.2 × 10^8^

ND: not detected in RC samples (day 22) due to the development of *G. candidum*; (-): not measured since the ripening bacteria were inoculated only on day 2. Culturability data are an average of two technical replicates.

## Data Availability

The datasets generated and/or analyzed during the current study are available in the Data INRAE repository, https://doi.org/10.57745/QRDUAE.

## Supplementary Materials

The following supporting information can be downloaded at: https://www.mdpi.com/article/10.3390/foods13121809/s1, Table S1: Aspect of the colonies of the ripening bacteria and yeasts in the cheese ecosystem; Tables S2 and S3: Culturability losses (log CFU.g^−1^) of fresh and ripened cheese ecosystems after freezing at −80 °C, −20 °C, and freeze-drying samples protected (MD, maltodextrin) and non-protected (S, saline solution); Table S4: Cell concentration (in CFU.g^−1^) of the microorganisms in the fresh and ripened cheese samples before stabilization; Table S5: Culturability losses (log CFU.g^−1^) after 1 month of storage of the fresh cheese ecosystem samples protected (MD, maltodextrin) and non-protected (S, saline solution), stabilized and stored frozen (−80 °C and −20 °C), and freeze-dried (4 °C and 25 °C); Table S6: Culturability losses (log CFU.g^−1^) after 1 month of storage of the ripened cheese ecosystem samples protected (MD, maltodextrin) and non-protected (S, saline solution), stabilized and stored frozen (−80 °C and −20 °C), and freeze-dried (4 °C and 25 °C); Figure S1: Glass transition temperatures of freeze-concentrated samples of fresh (FC, blue) and ripened (RC, red) cheese obtained from the first derivatives of the DSC heat flow curves. MD: maltodextrin (light color); S: saline solution (dark color); Figure S2: Thermal events observed by DSC in freeze-dried samples of fresh (FC, blue) and ripened (RC, red) cheese during (a) cooling from 40 to 0 °C and (b) heating (0 to 100 °C). Each subpanel enables the visual evaluation of the behavior of freeze-dried fresh and ripened samples with maltodextrin (MD, light color) or saline solution (S, dark color).
