# Supplementary material for: Investigation of Freezing and Freeze-Drying for Preserving and Re-Using a Whole Microbial Cheese Community
Source: Foods. 2024 Jun 8;13(12):1809. doi: 10.3390/foods13121809 (PMC11202935; doi:10.3390/foods13121809)
Supplement: Supplementary file 1 [file foods-13-01809-s001.zip › foods-3018406-supplementary.pdf]

Journal Foods

Supplementary Materials

Investigation of freezing and freeze-drying for preserving and re-using a whole microbial cheese community

Wenfan Cao, Stéphanie Passot, Françoise Irlinger and Fernanda Fonseca

Université Paris-Saclay, INRAE, AgroParisTech, UMR SayFood, F-911230 Palaiseau, France

Corresponding author: Fernanda Fonseca: Fernanda.fonseca@inrae.fr

Tables

Table S1. Aspect of the colonies of the ripening bacteria and yeasts in the cheese ecosystem.

	Picture of colonies	Strain	Appearance of colony (Petri dish)
Ripening bacteria<br><br>BHI agar<br>Amphotericin B<br>(50 mg.L-1)	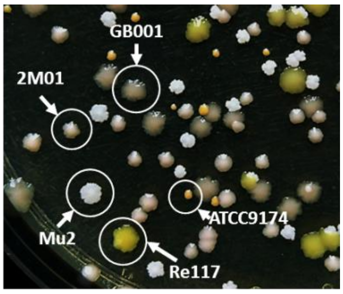	*G. arilaitensis* Re117	Yellow, shiny
		B. aurantiacum ATCC9174	Orange, small, shiny
		C. casei 2M01	Off-white, domed, shiny, with a nipple in the center of the colony
		H. alvei GB001	Ivory white, translucent, large, flat, shiny
		S. equorum Mu2	Titanium white, flat
Ripening yeasts<br><br>YEGC agar<br>TTC (10 mg.L-1)	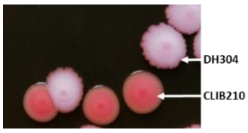	*D. hansenii* DH304	Domed, matt, pinkish-white, with a nipple in the center of the colony
		K. lactis CLIB210	Domed, shiny, red in the center, translucent at the edge
	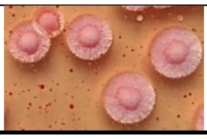	*G. candidum* ATCC204307	Filamentous, pinkish

BHI: brain heat infusion; YEGC: yeast extract glucose chloramphenicol; TTC: 2,3,5-Triphenyltetrazolium chloride.

Table S2. Culturability losses (log CFU.g^{-1}) of fresh and ripened cheese ecosystems after freezing at -80 °C, -20 °C and freeze-drying samples protected (MD, maltodextrin) and non-protected (S, saline solution).

	**Freezing -80 °C**				**Freezing -20 °C**				**Freeze-drying**			
	**Fresh cheese**		**Ripened cheese**		**Fresh cheese**		**Ripened cheese**		**Fresh cheese**		**Ripened cheese**	
**Strain**	**MD**	**S**	**MD**	**S**	**MD**	**S**	**MD**	**S**	**MD**	**S**	**MD**	**S**
G. arilaitensis	-0,40^{a}	-0,42^{ab}	-0,59^{a}	-0,74^{ab}	-0,88^{abcd}	-1,11^{abcd}	-0,69^{a}	-0,85^{abc}	-1,51^{bcd}	-1,67^{cd}	-1,34^{abcd}	-2,33^{d}
IQR	0,74	1,03	0,08	0,20	0,94	1,71	0,16	0,11	0,70	1,19	0,26	0,45
B. aurantiacum	-0,14^{ab}	-0,14^{abc}	-0,10^{a}	-0,25^{ab}	-0,77^{abc}	-0,77^{bcd}	-0,22^{abc}	-0,47^{abc}	-1,21^{d}	-1,55^{d}	-0,66^{bcd}	-0,81^{cd}
IQR	0,30	0,44	0,04	0,07	0,44	0,82	0,07	0,07	0,45	0,74	0,31	0,20
C. casei	-0,36^{abcd}	-0,22^{abc}	0,00^{a}	-0,10^{ab}	-2,12^{ef}	-2,48^{ef}	-0,11^{ab}	-0,48^{bcde}	-1,51^{cdef}	-1,56^{ef}	-1,51^{def}	-1,95^{f}
IQR	0,65	0,71	0,02	0,10	1,20	1,38	0,08	0,11	0,24	0,60	0,24	0,12
H. alvei	-0,05^{ab}	-0,03^{ab}	0,00^{a}	-0,19^{ab}	-0,05^{ab}	-0,30^{b}	-0,23^{ab}	-0,40^{bc}	-1,13^{cd}	-1,30^{cd}	-1,56^{d}	-2,11^{d}
IQR	0,15	0,15	0,06	0,07	0,13	0,40	0,17	0,17	0,19	0,12	0,10	0,27
S. equorum	-0,10^{ab}	-0,06^{ab}	-0,10^{ab}	-0,05^{a}	-0,05^{ab}	-0,22^{abc}	-0,25^{bcd}	-0,20^{abc}	-0,71^{de}	-0,85^{e}	-0,73^{cde}	-0,30^{bcd}
IQR	0,14	0,15	0,02	0,04	0,18	0,23	0,05	0,14	0,09	0,17	0,15	0,34
K. lactis	0,00^{a}	-0,02^{a}	ND	ND	-0,82^{a}	-1,16^{a}	ND	ND	-0,34^{a}	-0,66^{a}	ND	ND
IQR	0,13	0,20			0,71	1,13			0,48	0,48		
D. hansenii	0,00^{a}	0,00^{a}	ND	ND	-0,22^{ab}	-0,25^{abc}	ND	ND	-0,83^{c}	-0,59^{bc}	ND	ND
IQR	0,15	0,20			0,17	0,45			0,10	0,42		
G. candidum	-0,05^{a}	-0,12^{a}	-0,13^{a}	-0,33^{b}	-0,04^{a}	-0,13^{a}	-0,11^{a}	-0,15^{a}	-0,67^{b}	-1,92^{b}	-0,43^{b}	-0,67^{b}
IQR	0,03	0,11	0,06	0,06	0,05	0,04	0,10	0,12	0,66	0,80	0,06	0,05
L. lactis (S3+/S3-)	-0,01^{a}	-0,09^{ab}	-0,12^{ab}	-0,19^{abc}	-0,11^{ab}	-0,42^{abc}	-0,47^{abc}	-0,60^{bcd}	-0,62^{cde}	-0,76^{de}	-0,66^{de}	-0,86^{e}
IQR	0,07	0,15	0,09	0,09	0,17	0,18	0,25	0,31	0,11	0,15	0,32	0,30

Data presented are medians of three biological replicates and two technical replicates; IQR: interquartile ranges. Superscript letters (a, b, c, d, e, f) represent statistical differences (at the 95 % confidence level) for each micro-organism between all conditions: production step (fresh and ripened), formulation (S or MD) and stabilization conditions (freezing and freeze-drying). ND: not detected in RC samples (Day 22) due to the development of *G. candidum*.

Table S3. Culturability losses (log CFU.g^{-1}) of fresh and ripened cheese ecosystems after freezing at -80 °C, -20 °C and freeze-drying samples protected (MD, maltodextrin) and non-protected (S, saline solution).

	**Freezing -80 °C**				**Freezing -20 °C**				**Freeze-drying**			
	**Fresh cheese**		**Ripened cheese**		**Fresh cheese**		**Ripened cheese**		**Fresh cheese**		**Ripened cheese**	
**Strain**	**MD**	**S**	**MD**	**S**	**MD**	**S**	**MD**	**S**	**MD**	**S**	**MD**	**S**
G. arilaitensis	-0,40 ^{a}	-0,42 ^{a}	-0,59 ^{c}	-0,74 ^{d}	-0,88 ^{ab}	-1,11 ^{a}	-0,69 ^{b}	-0,85 ^{c}	-1,51 ^{d}	-1,67 ^{c}	-1,34 ^{b}	-2,33 ^{c}
IQR	0,74	1,03	0,08	0,20	0,94	1,71	0,16	0,11	0,70	1,19	0,26	0,45
B. aurantiacum	-0,14 ^{a}	-0,14 ^{a}	-0,10 ^{ab}	-0,25 ^{c}	-0,77 ^{ab}	-0,77 ^{a}	-0,22 ^{a}	-0,47 ^{bc}	-1,21 ^{cd}	-1,55 ^{bc}	-0,66 ^{a}	-0,81 ^{ab}
IQR	0,30	0,44	0,04	0,07	0,44	0,82	0,07	0,07	0,45	0,74	0,31	0,20
C. casei	-0,36 ^{a}	-0,22 ^{a}	0,00 ^{a}	-0,10 ^{ab}	-2,12 ^{b}	-2,48 ^{a}	-0,11 ^{a}	-0,48 ^{bc}	-1,51 ^{cd}	-1,56 ^{bc}	-1,51 ^{b}	-1,95 ^{c}
IQR	0,65	0,71	0,02	0,10	1,20	1,38	0,08	0,11	0,24	0,60	0,24	0,12
H. alvei	-0,05 ^{a}	-0,03 ^{a}	0,00 ^{ab}	-0,19 ^{bc}	-0,05 ^{ab}	-0,30 ^{a}	-0,23 ^{a}	-0,40 ^{ab}	-1,13 ^{bcd}	-1,30 ^{abc}	-1,56 ^{b}	-2,11 ^{c}
IQR	0,15	0,15	0,06	0,07	0,13	0,40	0,17	0,17	0,19	0,12	0,10	0,27
S. equorum	-0,10 ^{a}	-0,06 ^{a}	-0,10 ^{b}	-0,05 ^{a}	-0,05 ^{a}	-0,22 ^{a}	-0,25 ^{ab}	-0,20 ^{ab}	-0,71 ^{ab}	-0,85 ^{ab}	-0,73 ^{a}	-0,30 ^{a}
IQR	0,14	0,15	0,02	0,04	0,18	0,23	0,05	0,14	0,09	0,17	0,15	0,34
K. lactis	0,00 ^{a}	-0,02 ^{a}	ND	ND	-0,82 ^{ab}	-1,16 ^{a}	ND	ND	-0,34 ^{a}	-0,66 ^{a}	ND	ND
IQR	0,13	0,20			0,71	1,13			0,48	0,48		
D. hansenii	0,00 ^{a}	0,00 ^{a}	ND	ND	-0,22 ^{ab}	-0,25 ^{a}	ND	ND	-0,83 ^{abc}	-0,59 ^{ab}	ND	ND
IQR	0,15	0,20			0,17	0,45			0,10	0,42		
G. candidum	-0,05 ^{a}	-0,12 ^{a}	-0,13 ^{b}	-0,33 ^{d}	-0,04 ^{a}	-0,13 ^{a}	-0,11 ^{a}	-0,15 ^{a}	-0,67 ^{abc}	-1,92 ^{abc}	-0,43 ^{a}	-0,67 ^{a}
IQR	0,03	0,11	0,06	0,06	0,05	0,04	0,10	0,12	0,66	0,80	0,06	0,05
L. lactis (S3+/S3-)	-0,01 ^{a}	-0,09 ^{a}	-0,12 ^{bc}	-0,19 ^{bc}	-0,11 ^{ab}	-0,42 ^{a}	-0,47 ^{ab}	-0,60 ^{ab}	-0,62 ^{a}	-0,76 ^{a}	-0,66 ^{a}	-0,86 ^{b}
IQR	0,07	0,15	0,09	0,09	0,17	0,18	0,25	0,31	0,11	0,15	0,32	0,30

Data presented are medians of three biological replicates and two technical replicates; IQR: interquartile ranges. Superscript letters (a, b, c, d, e, f) represent statistical differences (at the 95 % confidence level) among micro-organisms, for each production/stabilization condition (column): production step (fresh and ripened), formulation (S or MD) and stabilization conditions (freezing and freeze-drying). ND: not detected in RC samples (Day 22) due to the development of *G. candidum*.

Table S4. Cell concentration (in CFU.g^{-1}) of the micro-organisms in the fresh and ripened cheese samples before stabilization.

	Cell concentration (CFU.g^{-1})	
	**Fresh cheese**	**Ripened cheese**
G. arilaitensis	7.9×10^5	2.97×10^7
IQR	2.19×10^5	2.07×10^7
B. aurantiacum	3.40×10^5	5.66×10^7
IQR	1.85×10^5	8.57×10^6
C.casei	2.09×10^6	1.58×10^9
IQR	9.48×10^5	7.83×10^7
H. alvei	1.31×10^6	1.71×10^8
IQR	3.05×10^5	6.96×10^7
S. equorum	1.36×10^6	9.07×10^7
IQR	1.96×10^5	5.90×10^7
K. lactis	8.60×10^6	$2.50.10^5$*
IQR	9.75×10^5	1.50×10^5
D. hansenii	1.44×10^7	1.77×10^6
IQR	$5.40.10^6$	1.83×10^6
G. candidum	8.80×10^4	$1.01.10^8$
IQR	6.75×10^5	2.16×10^7
L. lactis (S3+/S3-)	4.49×10^9	4.20×10^8
IQR	2.37×10^9	1.56×10^8

Data presented are medians of three biological replicates and two technical replicates; IQR: interquartile ranges. * *K. lactis* was detected in only two biological replicates.

Table S5. Culturability losses (log CFU.g^{-1}) after 1-month storage of the fresh cheese ecosystem samples protected (MD, maltodextrin) and non-protected (S, saline solution), stabilized and stored frozen (-80 °C and -20 °C) and freeze-dried (4 °C and 25 °C).

	**Frozen -80 °C**		**Frozen -20 °C**		**Freeze-dried 4 °C**		**Freeze-dried 25 °C**	
**Strain**	**MD**	**S**	**MD**	**S**	**MD**	**S**	**MD**	**S**
G. arilaitensis	-0,18 ^{a}	-0,09 ^{a}	-0,28 ^{a}	-1,00 ^{ab}	-1,79 ^{bc}	-0,77 ^{ab}	-2,09 ^{c}	-1,93 ^{bc}
IQR	0,12	0,37	0,14	0,83	0,57	0,52	0,31	0,80
B. aurantiacum	-0,10 ^{a}	0,00 ^{a}	-0,18 ^{a}	-0,09 ^{ab}	-2,33 ^{bc}	-0,97 ^{ab}	-3,06 ^{b}	-2,26 ^{bc}
IQR	0,11	0,06	0,06	0,18	0,79	0,61	0,58	0,76
C.casei	-0,12 ^{a}	-0,26 ^{a}	-1,00 ^{bc}	-0,78 ^{b}	-1,26 ^{bc}	-0,49 ^{a}	-2,21 ^{d}	-1,63 ^{cd}
IQR	0,07	0,24	0,19	0,15	0,40	0,31	0,38	0,33
H. alvei	-0,02 ^{a}	-0,24 ^{bc}	-0,07 ^{bc}	-0,80 ^{c}	-1,41 ^{d}	-0,71 ^{c}	-3,69 ^{e}	-3,39 ^{de}
IQR	0,01	0,12	0,10	0,43	0,36	0,29	0,09	0,31
S. equorum	-0,01 ^{a}	-0,25 ^{ab}	-0,08 ^{a}	-0,08 ^{a}	-1,57 ^{cd}	-0,69 ^{bc}	-2,19 ^{e}	-1,25
IQR	0,12	0,03	0,20	0,34	0,13	0,34	0,19	0,23
K. lactis	0,00 ^{ab}	0,00 ^{a}	-2,51 ^{cd}	-2,36 ^{d}	-0,57 ^{bc}	-0,01 ^{ab}	-0,84 ^{cd}	-0,65 ^{cd}
IQR	0,16	0,29	1,54	1,19	0,37	0,07	0,19	0,20
D. hansenii	-0,42 ^{a}	-0,09 ^{a}	-0,36 ^{a}	-0,04 ^{a}	-0,60 ^{a}	-0,08 ^{a}	-1,04 ^{a}	-0,84 ^{a}
IQR	0,16	0,26	0,39	0,49	0,32	0,55	0,46	0,66
G. candidum	-0,02 ^{a}	-0,20 ^{ab}	-0,35 ^{abc}	-1,07 ^{c}	-0,36 ^{abc}	-0,23 ^{ab}	-0,75 ^{abc}	-0,87 ^{bc}
IQR	0,12	0,15	0,28	0,31	0,22	0,07	0,29	0,44
L. lactis	-0,13 ^{ab}	-0,05 ^{a}	0,00 ^{a}	-0,25 ^{ab}	-0,35 ^{bc}	-0,07 ^{a}	-0,68 ^{c}	-0,70 ^{c}
IQR	0,12	0,05	0,12	0,18	0,05	0,05	0,12	0,21

Data presented are medians three biological replicates and two technical replicates; IQR: interquartile ranges. Superscript letters (a, b, c, d) represent statistical differences (at the 95 % confidence level) for each micro-organism between all conditions: formulation (S or MD) and storage conditions (-80 °C and -20 °C, frozen samples; 4 °C and 25 °C, freeze-dried samples)

Table S6. Culturability losses (log CFU.g^{-1}) after 1-month storage of the ripened cheese ecosystem samples protected (MD, maltodextrin) and non-protected (S, saline solution), stabilized and stored frozen (-80 °C and -20 °C) and freeze-dried (4 °C and 25 °C).

	**Frozen -80 °C**		**Frozen -20 °C**		**Freeze-dried 4 °C**		**Freeze-dried 25 °C**	
**Strain**	**MD**	**S**	**MD**	**S**	**MD**	**S**	**MD**	**S**
G. arilaitensis	0,00 ^{a}	-0,01 ^{a}	-0,75 ^{b}	-0,54 ^{b}	-0,86 ^{b}	-0,98 ^{b}	-2,84 ^{c}	-1,70 ^{c}
IQR	0,06	0,05	0,33	0,31	0,11	0,14	0,32	0,11
B. aurantiacum	-0,10 ^{ab}	-0,06 ^{a}	-0,12 ^{b}	-0,17 ^{ab}	-0,98 ^{c}	-1,37 ^{c}	-2,25 ^{d}	-2,11 ^{d}
IQR	0,12	0,04	0,06	0,09	0,20	0,35	0,21	0,61
C. casei	-0,04 ^{a}	-0,03 ^{a}	-0,09 ^{a}	-0,37 ^{b}	-1,82 ^{c}	-1,47 ^{c}	-2,90 ^{d}	-3,81 ^{d}
IQR	0,02	0,08	0,11	0,11	0,18	0,39	0,15	0,38
H. alvei	-0,01 ^{ab}	0,00 ^{a}	-0,09 ^{b}	-0,57 ^{c}	-0,74 ^{c}	-0,68 ^{c}	-2,48 ^{d}	-2,31 ^{d}
IQR	0,12	0,00	0,01	0,10	0,18	0,51	0,37	0,31
S. equorum	-0,04 ^{a}	-0,15 ^{ab}	0,00 ^{a}	-0,01 ^{a}	-0,62 ^{bcd}	-0,50 ^{bc}	-1,51 ^{cd}	-1,60 ^{d}
IQR	0,04	0,07	0,03	0,06	0,21	0,40	0,32	0,59
G. candidum	-0,10 ^{a}	-0,14 ^{a}	-0,04 ^{a}	-0,08 ^{a}	-0,11 ^{a}	-0,11 ^{a}	-0,33 ^{a}	-0,19 ^{a}
IQR	0,08	0,05	0,11	0,09	0,07	0,11	0,14	0,07
L. lactis	-0,02 ^{ab}	-0,01 ^{ab}	0,00 ^{a}	0,00 ^{a}	-0,38 ^{bc}	-0,31 ^{abc}	-0,82 ^{c}	-1,06 ^{c}
IQR	0,09	0,10	0,00	0,08	0,14	0,36	0,22	0,40

Data presented are medians three biological replicates and two technical replicates; IQR: interquartile ranges.

Superscript letters (a, b, c, d) represent statistical differences (at the 95 % confidence level) for each micro-organism between all conditions: formulation (S or MD) and storage conditions (-80 °C and -20 °C, frozen samples; 4 °C and 25 °C, freeze-dried samples)

Figures

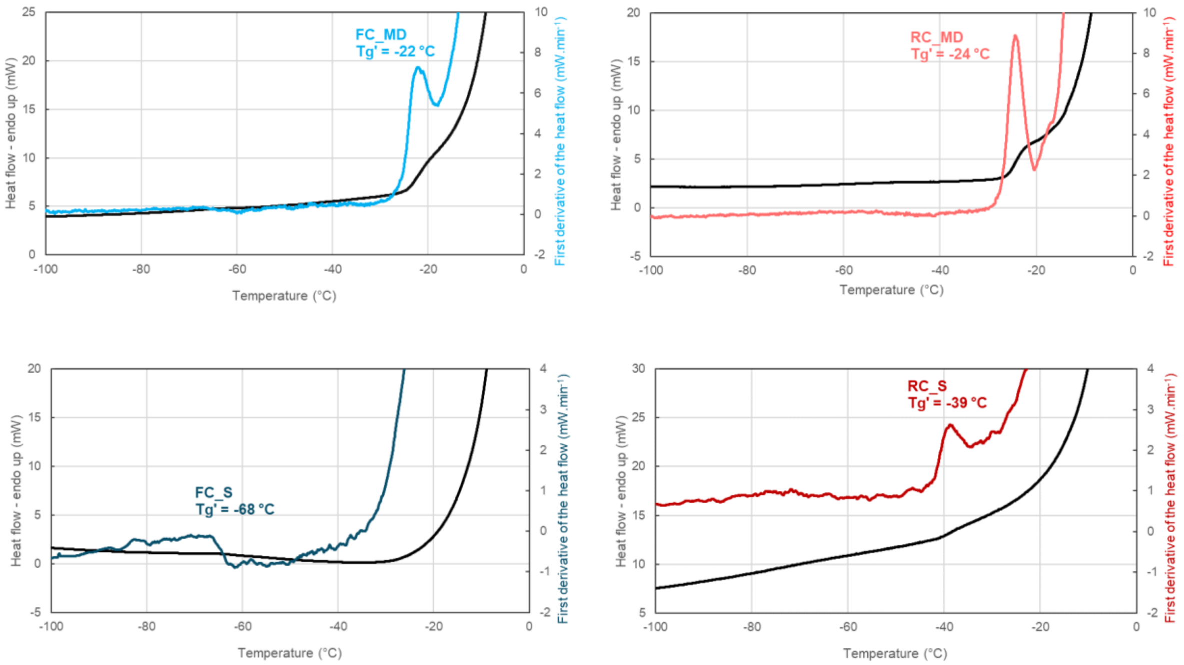

Figure S1. Glass transition temperatures of freeze-concentrated samples of fresh (FC, blue) and ripened (RC, red) cheese obtained from the first derivatives of DSC the heat flow curves. MD: maltodextrin (light color); S: saline solution (dark color).

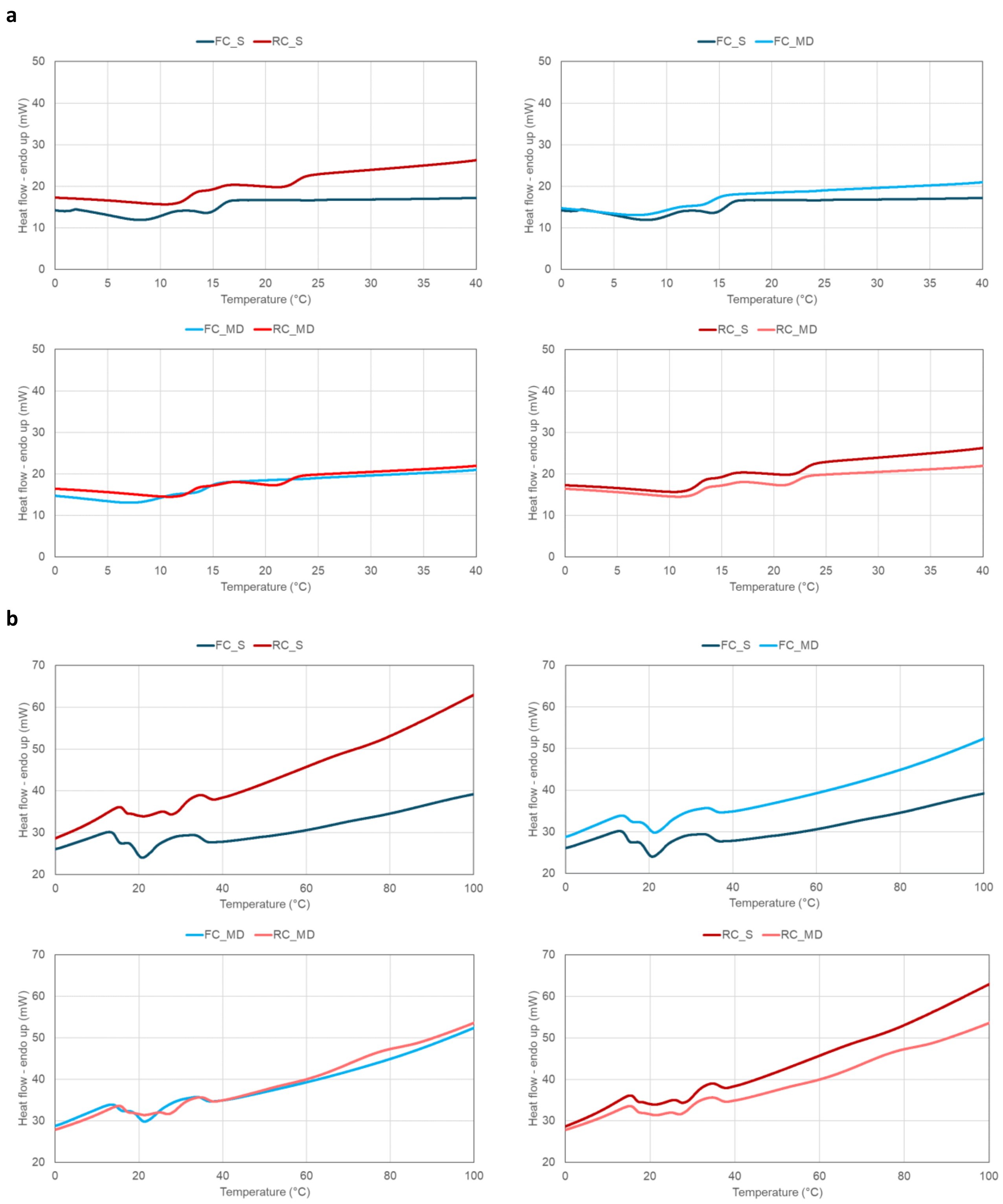

Figure S2. Thermal events observed by DSC in freeze-dried samples of fresh (FC, blue) and ripened (RC, red) cheese during (a) cooling from 40 to 0 °C and (b) heating (0 to 100 °C). Each subpanel enables the visual evaluation of the behavior of freeze-dried fresh and ripened samples with maltodextrin (MD, light color) or saline solution (S, dark color).
